# Discovery of *TBX20* as a Novel Gene Underlying Atrial Fibrillation

**DOI:** 10.3390/biology12091186

**Published:** 2023-08-30

**Authors:** Ning Li, Yan-Jie Li, Xiao-Juan Guo, Shao-Hui Wu, Wei-Feng Jiang, Dao-Liang Zhang, Kun-Wei Wang, Li Li, Yu-Min Sun, Ying-Jia Xu, Yi-Qing Yang, Xing-Biao Qiu

**Affiliations:** 1 Department of Cardiology, Putuo Hospital, Shanghai University of Traditional Chinese Medicine, Shanghai 200062, China; frankleelx@163.com; 2 Department of Cardiology, Shanghai Chest Hospital, School of Medicine, Shanghai Jiao Tong University, Shanghai 200030, China; liyanjie6666@126.com (Y.-J.L.); wushaohui18@163.com (S.-H.W.); wf.jiang@hotmail.com (W.-F.J.); 3 Department of Cardiology, Shanghai Fifth People’s Hospital, Fudan University, Shanghai 200240, China; guo.xiaojuan@foxmail.com (X.-J.G.); xuyingjia@5thhospital.com (Y.-J.X.); 4 Center for Complex Cardiac Arrhythmias of Minhang District, Shanghai Fifth People′s Hospital, Fudan University, Shanghai 200240, China; 5 Cardiac Arrhythmia Center, Fuwai Hospital, Chinese Academy of Medical Sciences, Shenzhen 518057, China; xkzhangdaoliang@126.com; 6 Department of Cardiology, Tongji Hospital, Tongji University School of Medicine, Shanghai 200065, China; wkw2012518@163.com; 7 Key Laboratory of Arrhythmias, Ministry of Education of China, Tongji University School of Medicine, Shanghai 200092, China; lilirz@tongji.edu.cn; 8 Department of Cardiology, Shanghai Jing’an District Central Hospital, Fudan University, Shanghai 200040, China; sunyumin@fudan.edu.cn; 9 Cardiovascular Research Laboratory, Shanghai Fifth People’s Hospital, Fudan University, Shanghai 200240, China; 10 Central Laboratory, Shanghai Fifth People’s Hospital, Fudan University, Shanghai 200240, China

**Keywords:** cardiac arrhythmia, atrial fibrillation, medical genetics, molecular biology, linkage analysis, transcriptional regulation, TBX20, biological assay

## Abstract

**Simple Summary:**

Atrial fibrillation (AF), the most prevalent sustained dysrhythmia, is accountable for substantial mortality and morbidity. Accumulating convincing evidence highlights the predominant roles of heritable components in the initiation and maintenance of AF. Here, through pan-genomic genotyping with genetic markers followed by a genetic linkage study in an AF family, a novel AF-causing locus was located at human chromosome 7p14.2–p14.3. An exome-wide sequence assay unveiled that, at the defined locus, the mutation in *TBX20*, NM_001077653.2: c.695A>G; p.(His232Arg), was solely co-segregated with AF in the family. Additionally, a Sanger sequencing assay of *TBX20* in another AF family uncovered a novel mutation, NM_001077653.2: c.862G>C; p.(Asp288His). Neither of the two mutations were found in 600 control persons. Functional investigations demonstrated that the two mutations both significantly reduced the transactivation of the target gene *KCNH2* and the ability to bind the promoter of *KCNH2*, while they had no effect on the nuclear distribution of TBX20. These findings strongly indicate that *TBX20* is a new AF-predisposing gene, shedding light on the mechanism underlying AF and suggesting clinical significance for the individually tailored treatment of AF.

**Abstract:**

Atrial fibrillation (AF), the most prevalent type of sustained cardiac dysrhythmia globally, confers strikingly enhanced risks for cognitive dysfunction, stroke, chronic cardiac failure, and sudden cardiovascular demise. Aggregating studies underscore the crucial roles of inherited determinants in the occurrence and perpetuation of AF. However, due to conspicuous genetic heterogeneity, the inherited defects accounting for AF remain largely indefinite. Here, via whole-genome genotyping with genetic markers and a linkage assay in a family suffering from AF, a new AF-causative locus was located at human chromosome 7p14.2-p14.3, a ~4.89 cM (~4.43-Mb) interval between the markers D7S526 and D7S2250. An exome-wide sequencing assay unveiled that, at the defined locus, the mutation in the *TBX20* gene, NM_001077653.2: c.695A>G; p.(His232Arg), was solely co-segregated with AF in the family. Additionally, a Sanger sequencing assay of *TBX20* in another family suffering from AF uncovered a novel mutation, NM_001077653.2: c.862G>C; p.(Asp288His). Neither of the two mutations were observed in 600 unrelated control individuals. Functional investigations demonstrated that the two mutations both significantly reduced the transactivation of the target gene *KCNH2* (a well-established AF-causing gene) and the ability to bind the promoter of *KCNH2*, while they had no effect on the nuclear distribution of TBX20. Conclusively, these findings reveal a new AF-causative locus at human chromosome 7p14.2-p14.3 and strongly indicate *TBX20* as a novel AF-predisposing gene, shedding light on the mechanism underlying AF and suggesting clinical significance for the allele-specific treatment of AF patients.

## 1. Introduction

Atrial fibrillation (AF) is believed to be the most common cardiac dysrhythmia encountered in clinics, occurring in ~2% of the general population around the globe [1,2]. The prevalence of this cardiac rhythm disturbance increases dramatically with advancing age, increasing from approximately 1% in patients aged <60 years up to 12% in those 75 to 84 years old [1]. The lifetime risks of developing AF are ~25% in individuals after the age of 40 years and ~37% in those >55 years old [3,4]. Based on the reported global burden of disease, AF affects more than 43 million persons globally [5]. Given that approximately one-third of individuals with subclinical or silent AF are asymptomatic and likely undiagnosed, the actual prevalence of AF is obviously underestimated [6,7,8]. AF may markedly reduce the cardiac output with adverse hemodynamic consequences [1], contributing to a downgraded health-correlated quality of life [9,10,11,12], reduced exercise tolerance [13,14,15], impaired cognitive function and even dementia [16,17,18,19,20,21], ischemic cerebral stroke or systemic embolism [22,23,24,25], acute renal injury or chronic kidney disease [26,27,28], myocardial infarction [29,30,31], chronic/congestive heart failure [32,33,34], lethal ventricular arrhythmias [35], and premature cardiovascular death [36,37,38,39]. In fact, it has been reported that AF causes a 5-fold enhanced risk of stroke and is accountable for roughly one-third of all strokes, and, furthermore, AF-related stroke is associated with higher mortality compared with non-AF-associated stroke [1]. AF also confers a 3-fold enhanced risk for congestive cardiac failure as well as a 2-fold enhanced risk for dementia or demise [1]. Over the last decades, tremendous advancement has been achieved in the radical treatment of AF, especially in percutaneous catheter ablation procedures and cardiac Cox maze surgeries, which successfully restore the sinus rhythm in most AF patients [40,41,42,43,44]. However, the recurrence events of AF subsequent to catheter-based ablation or cardiac surgical therapy remain a major clinical challenge [45,46,47,48,49], irrespective of the decreasing but formidable periprocedural complications, encompassing pulmonary vein stenosis, systemic thromboembolism, esophageal injury, atrial-esophageal fistula, and cardiac perforation/tamponade [50]. Obviously, AF accounts for considerable mortality, substantial morbidity, and vast socioeconomic encumbrance [1]. Despite the paramount clinical importance, the pathophysiological mechanisms that initiate and perpetuate AF remain incompletely understood.

Accumulating epidemiological evidence strongly indicates that the etiologies responsible for the development and maintenance of AF are extremely diverse and complex, and both environmental/non-genetical risk factors and heritable causative components may give rise to AF [2,51,52,53]. The already ascertained environmental/modifiable factors predisposing to AF encompass hypertrophic/dilated cardiomyopathy, coronary heart disease/acute myocardial infarction, valvular heart disease, essential hypertension, hyperthyroidism, diabetes mellitus, obstructive sleep apnea/hypopnea syndrome, an imbalanced cardiac autonomic nervous system, peri-atrial inflammation, β-thalassemia, metabolic disorder, obesity, smoking, alcohol consumption, and sedentary lifestyles [2,51,54,55,56,57,58,59,60,61].

However, aggregating investigations have convincingly substantiated that genetic determinants exert critical roles in the initiation and perpetuation of AF, especially for idiopathic/familial AF, and, to date, a great number of rare AF-causing variations in >60 genes have been causally related to AF, amidst which the overwhelming majority encode ion channel subunits, myocardial structural proteins, signaling molecules, cardiac transcription factors, and connexins [52,53,62,63,64,65,66,67,68,69,70,71,72,73,74]. In addition, pan-genomic association research has revealed that common variants at ~140 genetic loci are implicated with enhanced vulnerability to AF, though merely a small fraction of these recognized variants have been experimentally validated to be pathogenic for AF thus far [52]. Notably, both rare mutations and common variations in the *KCNH2* gene, which codes for the α subunit of the voltage-gated K^+^ channel subfamily H member 2 and which has its expression transactivated by TBX20 [75], have been causally involved in AF [76,77,78]. Nevertheless, the genetic defects underpinning AF remain largely indefinite because of conspicuous genetic heterogeneity.

In this investigation, to discover a new gene accountable for AF, a large pedigree suffering from AF was prospectively enlisted. Through whole-genome screening with microsatellite DNA markers and genetic linkage examination, a new AF-causative locus was located at human chromosome 7p14.2-p14.3. Sequence analysis of the genes at the defined locus in the pedigree with AF followed by functional exploration indicates that *TBX20* is a novel gene causative for AF. These findings throw new light on the molecular pathogenesis of AF.

## 2. Materials and Methods

### 2.1. Recruitment and Clinical Investigation of Subjects

In this research, a 36-member family (arbitrarily termed Family 1) and a 39-member family (arbitrarily designated as Family 2), both spanning four generations with a high incidence of AF, were discovered in a Han-ethnicity population in China. The living members from Family 1 and Family 2 and another cohort of 216 unrelated cases suffering from idiopathic AF were recruited. A total of 600 unrelated healthy people with a negative family history of AF were employed as controls. A comprehensive clinical evaluation was implemented in all study participants by cardiologists, including thorough reviews of personal histories and medical histories (encompassing symptoms such as syncope and palpitation, cardiovascular diseases diagnosed previously, and the medications prescribed), detailed physical examination, routine laboratory tests, a transthoracic echocardiogram, and a 12-lead electrocardiogram. When indicated, a 48-hour ambulatory electrocardiogram and cardiac electrophysiological examination were carried out. The medical records in hospitals were examined for the members of Family 1 and Family 2 who had died. Clinical diagnosis and categorization (idiopathic/secondary or paroxysmal/persistent/permanent) of AF were made as described elsewhere [1,79]. The current investigation was completed in compliance with the tenets stated in the Declaration of Helsinki, and the protocols applied to this research were approved by the institutional ethical committee at the Chest Hospital, School of Medicine, Shanghai Jiao Tong University, China (ethical approval code: KS1101). Prior to recruitment for the research, the adult participants and the legal representatives of the adolescents signed the informed consent. A peripheral blood specimen (approximately 6.0 mL) was collected in an EDTA-anticoagulation tube (BD, Franklin Lakes, NJ, USA) from each research participant, and a human blood DNA purification kit (Qiagen GmbH, Hilden, Germany) was applied to the isolation of genomic DNA from blood leukocytes of all research participants.

### 2.2. Whole-Genome Scan Using Markers and Linkage Assay

A whole-genome screening was completed in the 34 living members from Family 1 suffering from AF using linkage mapping sets (Applied Biosystems, Foster City, CA, USA), with a total of 398 fluorescently labeled polymorphic DNA markers/microsatellites, which were spaced at an even density of ~9 cM throughout the 22 human autosomes as described previously [80,81,82]. Multiplex amplification of three or four polymorphic microsatellite markers was implemented via polymerase chain reaction (PCR) utilizing a Taq DNA polymerase kit (Applied Biosystems, Foster City, CA, USA) on a PCR instrument (Bio-Rad, Hercules, CA, USA). The amplicons were separated by gel electrophoresis under genetic analysis equipment (ABI, USA) following the manufacturer’s protocol and genotyped utilizing the GeneScan software version 3.7 (Applied Biosystems, Foster City, CA, USA) and Genotyper version 3.7 (Applied Biosystems, Foster City, CA, USA). Linkage analysis as well as the computation of the scores of the 2-point logarithm of odds (LOD) was completed as previously described [80,81,82]. When a supportive score of two-point LOD was obtained for a marker on human chromosome 7, seven additional microsatellite markers were chosen to map finely. The average distance between two of the eight microsatellite markers was ~1.99 cM. Haplotypes of Family 1 with AF were generated to show the common chromosomal regions amongst the family members affected by AF and confine the recombinant chromosomal borders. Genotypes were achieved independently from phenotypes.

### 2.3. Sequence Analysis of the Genes Located at the Finely Mapped Locus

A whole-exome sequencing (WES) assay in two family members affected by AF (Family 1: III-1 and IV-1) and one healthy family member (Family 1: III-2) was completed as previously described [83,84,85]. Briefly, for a chosen family member, 2 µg of genomic DNA was utilized to prepare a genomic library with a DNA library construction kit (New England Biolabs, Ipswich, MA, USA) as per the manufacturer’s recommendations. An exome library was built by capturing the exome regions from a genomic library using the kit of Human All Exon V6 (Agilent Technologies, Santa Clara, CA, USA) as per the manufacturer’s manual. Exome libraries were sequenced under the HiSeq4000 instrument (Illumina, San Diego, CA, USA) according to the manual. Raw data from exome sequencing were processed with a standard pipeline (Illumina, San Diego, CA, USA) for calling bases and aligned to the human genome (build GRCh37/hg19) utilizing BWA. Sequence variations within targeted regions, including single-nucleotide variations and deletions/insertions, were identified using the SAMtools software version 1.0. The genomic sequence variations were annotated using the ANNOVAR software version 2015, and the variations with a population frequency of ≥1% in the database of gnomAD (http://gnomad-sg.org/, accessed again on 4 February 2022) were excluded. The non-synonymous variations as well as those generating alternate splicing sites or premature stop codons were subjected to further analysis, encompassing Sanger sequencing analysis of the entire coding regions as well as splicing junctions of the genes carrying potentially causative variations and co-segregation analysis in the AF family (Family 1). Once a gene harboring an AF-causing mutation was discovered in Family 1, a sequence assay of the same gene was conducted in Family 2 and another cohort of 216 index cases affected by AF as well as 600 unrelated healthy people who were employed as control persons. For a validated malicious mutation, the databases of gnomAD (http://gnomad-sg.org/ consulted again on 8 January 2022) and SNP (https://www.ncbi.nlm.nih.gov/snp/ consulted again on 8 January 2022) were retrieved to ascertain its novelty.

### 2.4. Production of Eukaryotic Gene Expression Vectors

The eukaryotic gene expression vector TBX20-pcDNA3.1 expressing wild-type human TBX20 (GenBank accession number: NM_001077653.2) was constructed as described elsewhere [86]. Each mutant-type TBX20-pcDNA3.1 vector was produced via site-targeted mutagenesis of wild-type TBX20-pcDNA3.1 utilizing a complementary pair of primers as well as a site-targeted mutagenesis system (Invitrogen, Carlsbad, CA, USA) and was verified through sequence analysis. A 900 bp genomic DNA sequence segment (from –700 to +200, with the transcriptional initial nucleotide position denoted as +1) of the human *KCNH2* gene (GenBank accession number: NC_000007.14) was acquired through PCR-based amplification of human genomic DNA using specific oligonucleotide primer pairs (forward primer: 5′-CACGCTAGCGCTCCTATGCAGAGCCCCAC-3′; backward primer: 5′-GTGCTCGAGCTGAGCGCGAGCCGCCCGCC-3′), subsequently cut with the restriction endonucleases of *Nhe*I (Thermo Fisher Scientific, Waltham, MA, USA) and *Xho*I (Thermo Fisher Scientific, Waltham, MA, USA), and finally subcloned into the pGL3-Basic plasmid (Promega, Madison, WI, USA) to generate the *KCNH2* promoter-driven firefly luciferase reporter vector (KCNH2-luc).

### 2.5. Cellular Vector Transfection Followed by Dual-Reporter Assay

HeLa cells were cultured routinely and seeded into a plate with 24 wells, 24 h prior to vector transfection with a lipofectamine reagent (Invitrogen, Carlsbad, CA, USA) as described previously [87]. Various amounts (varying from 25 ng to 400 ng) of wild-type TBX20-pcDNA3.1 vector were transfected to test its dose-dependent activation of KCNH2-luc (1.0 μg) in the presence of 40 ng of pRL-TK (Promega, Madison, WI, USA). To analyze the transactivation of the *KCNH2* promoter by TBX20, cells were transiently transfected with 100 ng of empty pcDNA3.1 vector or 100 ng of wild-type TBX20-pcDNA3.1 vector or 100 ng of mutant TBX20-pcDNA3.1 vector or 50 of wild-type TBX20-pcDNA3.1 vector plus 50 ng of empty pcDNA3.1 vector or 50 ng of wild-type TBX20-pcDNA3.1 vector plus 50 ng of mutant TBX20-pcDNA3.1 vector, together with 1.0 μg of the KCNH2-luc vector and 40 ng of the pRL-TK vector (Promega, USA). The pRL-TK vector expressing renilla luciferase (Promega, USA) was utilized to correct for transfection efficiency. The cells transfected with gene expression vectors were gathered and lysed 48 h post cellular transfection. The cellular lysates were collected into a 96-well microtiter plate (Greiner Bio-One GmbH, Frickenhausen, Germany). The dual-luciferase activities of the lysates were measured under the Infinite^®^ 200 PRO NanoQuant spectrophotometer (Tecan, Männedorf, Switzerland) using dual-luciferase reporter analysis kits (Promega, Madison, WI, USA) as per the manufacturers’ specifications. The activity values for the promoter of *KCNH2* were computed as ratios of firefly luciferase activity relative to renilla luciferase activity. Each transfection experiment for the reporter assay was repeated three times in triplicate.

### 2.6. Assay of Electrophoretic Mobility of Mutant TBX20

Cellular nuclear and cytoplasmic proteins were purified from the cultivated Hela cells that were transiently transfected with the wild-type or mutant-type TBX20 expression vector (TBX20-pcDNA3.1) by utilizing a cellular protein purification kit (Thermo Scientific, USA) as per the instructions. A 22 bp core DNA fragment (forward 5′-GCAGACAGGTGTGCCGGCGGCG-3′ and reverse 5′-CGCCGCCGGCACACCTGTCTGC-3′) within the *KCNH2* promoter, which harbors a highly conserved consensus binding site (i.e., 5′-AGGTGTG-3′) for the TBX family of transcription factors [88], was synthesized and labeled with biotin at both 5′ and 3′ ends. These biotinylated oligonucleotide probes (0.2 pmol) with a TBX20-binding site, which were annealed into double strands, were mixed with the purified nuclear proteins (5 μg) containing wild-type or mutant TBX20 protein and incubated in the binding buffer for 20 min. Notably, an unlabeled cold oligonucleotide probe (20 pmol) was pre-incubated as a competitor with the purified nuclear proteins for 10 min to increase the specificity of the binding. The protein–DNA complex was separated from the free oligonucleotide probes in 6% native polyacrylamide gel under a voltage of 100 V lasting for 1 h, transferred to a nylon membrane (Roche, Mannheim, Germany) under a constant electric current of 380 mA for 30 min, and subsequently photo-cross-linked through ultraviolet irradiation for 20 min. To evaluate the DNA-binding ability, the cross-linked protein–DNA complex was detected under the ImageQuant LAS 4000mini instrument (GE Healthcare, Chicago, IL, USA) with a chemiluminescent EMSA kit (Thermo Scientific, USA) following the manufacturer’s specifications.

### 2.7. Subcellular Distribution of TBX20 Mutants

Hela cells were cultivated in a 12-well plate, where a round-shape glass coverslip with a diameter of 14 mm was pre-placed at the bottom of each well. Cells at approximately 80% confluency were transiently transfected with 100 ng of the wild-type or mutant-type TBX20 expression vector (TBX20-pcDNA3.1). Cells were harvested 48 h post transient transfection, fixed with 4% paraformaldehyde for 15 min, and washed with a pre-cooled buffer in a shaker. The fix-treated cells were put into an antigen retrieval buffer, heated at 95 °C for 10 min, washed with the buffer, and then permeabilized using 0.5% TritonX-100 for 30 min. Thereafter, the cells fixed on a glass coverslip were blocked utilizing 3% BSA for 30 min and incubated with the rabbit anti-TBX20 primary antibody (Affinity Biosciences, Liyang, Jiangsu, China) diluted at 1:200 overnight at 4 °C, and then they were incubated with the secondary antibody-conjugated goat anti-rabbit Alexa-Flour 594 (Affinity Biosciences, Liyang, Jiangsu, China) for 1 h. Nuclear staining was completed with DAPI (Sigma-Aldrich, St. Louis, MO, USA) for 1 min. Finally, glass coverslips were mounted using ProLong^TM^ Glass Antifade Mountant (Invitrogen, Carlsbad, CA, USA) and sealed with a commercial sealant. The fluorescence images of cells were recorded and saved with confocal fluorescence microscopy (Leica Microsystems, Wetzlar, Germany).

### 2.8. Statistical Assessment

All statistical tests for this study were completed with SAS version 9.4 (SAS Institutes, Cary, NC, USA). Normally distributed continuous data were presented by the mean ± standard deviation. Categorical data were expressed with the number and proportion (n, %). For normally distributed continuous variables, an unpaired Student t-test was employed to test for differences between two groups. To compare categorical variables between two groups, a Fisher’s exact test or Chi-square test was employed as appropriate. Two-tailed *p* values < 0.05 indicated statistical difference.

## 3. Results

### 3.1. Phenotypic Characteristic Profiles of Study Participants

**Figure 1:**
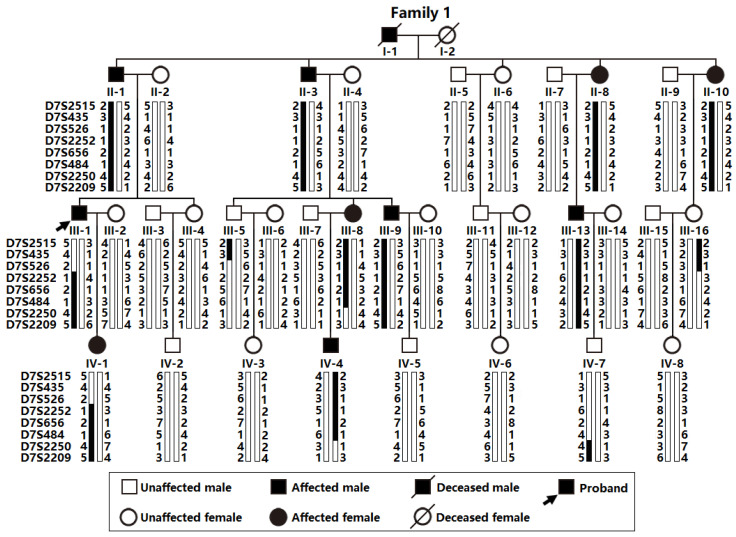
Haplotype analysis of microsatellite markers at chromosome 7p14.2-p14.3 in Family 1 suffering from atrial fibrillation. A vertical bar represents the chromosomal segment delimited via genotypic analysis using polymorphic genetic markers. The chosen eight markers covering the linked locus on chromosome 7p14.2-p14.3 are given to the left of the pedigree, and the family members’ genotypes (symbolized with Arabic numbers) for these microsatellite markers are exhibited next to the chromosomal bars. A blackened vertical bar denotes a haplotype segregating with atrial fibrillation, in contrast to an open one indicating a normal haplotype.

As illustrated in Figure 1, a 36-member pedigree spanning four generations affected by AF (Family 1), including 34 living members (17 female and 17 male individuals, with ages varying from 15 years to 70 years), was enlisted from the Chinese population of the Han race.

**Figure 2:**
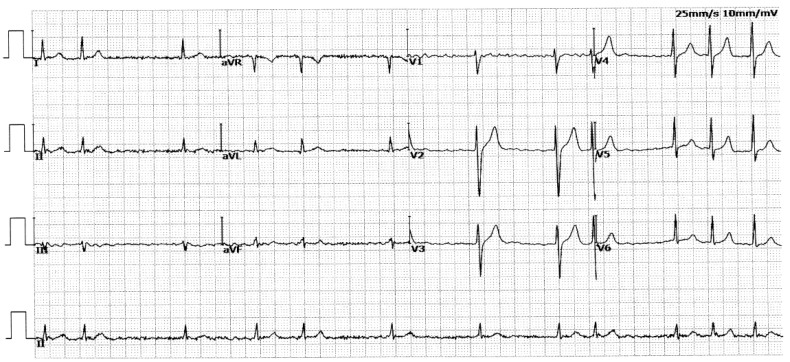
A typical electrocardiograph from the index patient (III-1) of Family 1. The representative electrocardiograph illustrates atrial fibrillation.

In this four-generation family (Family 1), in total there existed 11 members suffering from AF documented on the electrocardiograms, of whom the proband’s grandfather (I-1) died of an acute thromboembolic cerebral stroke when he was 61 years old. Among the 11 members affected by AF, 2 (II-8 and III-13) also had a congenital atrial septal defect, and the 9 others had no cardiac structural defects. All 25 unaffected members in Family 1 had a negative history of AF, with normal electrocardiograms and echocardiograms. The proband from Family 1 (III-1) was firstly diagnosed with AF during a routine physical examination at 28 years of age. A representative electrocardiograph of the proband from Family 1 (III-1) is displayed in Figure 2.

The phenotypic characteristics of the living members suffering from AF from Family 1 are summed in Table 1.

**Figure 3:**
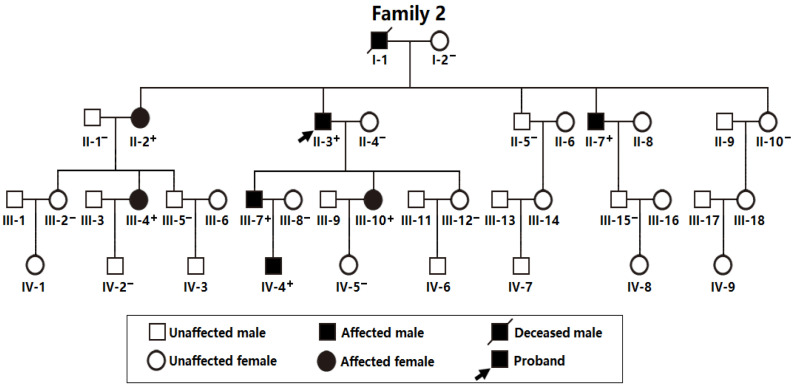
Pedigree of Family 2 suffering from atrial fibrillation. A “⁺” symbol denotes a heterozygote for the identified *TBX20* mutation of c.862G>C (p.D288H), in contrast to a “−” symbol denoting a homozygote for wild-type *TBX20*.

As exhibited in Figure 3, a 39-member family spanning four generations affected by AF (Family 2), including 38 living members (19 male individuals and 19 female individuals, with ages ranging between 8 years and 87 years), was enrolled from the Chinese population of Han ethnicity.

**Figure 4:**
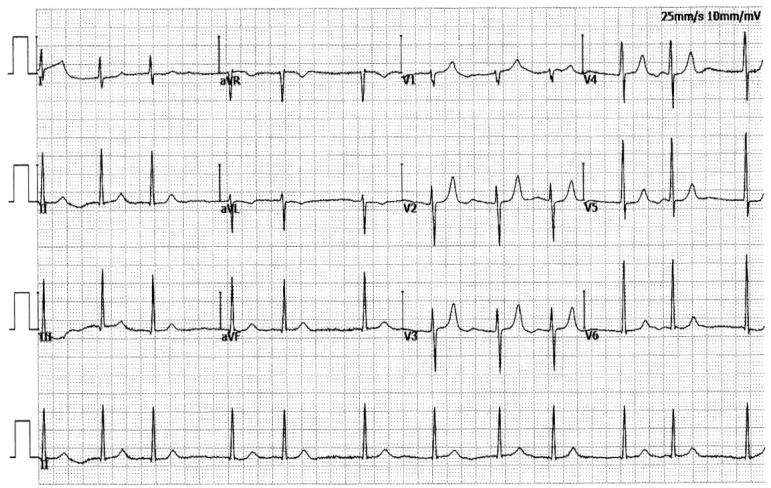
A typical electrocardiogram from the proband (II-3) of Family 2. The representative electrocardiogram documents atrial fibrillation.

In this four-generation family (Family 2), there were 8 members affected by AF documented on the surface electrocardiograms, of whom the proband’s grandfather (I-1) died of a recurrent ischemic cerebral stroke when he was 63 years old. Amongst the eight members affected by AF, one member (II-7) also had a congenital atrial septal defect, and the seven other members had no cardiac structural abnormalities. All 31 unaffected family members from Family 2 had no history of AF, with normal electrocardiograms and echocardiograms. The index case from Family 2 (II-3) was initially diagnosed with AF due to palpitation, fatigue, and dizziness at 25 years of age. A representative electrocardiogram of the index case from Family 2 (II-3) is provided in Figure 4.

The phenotypic characteristic information of the AF members available from Family 2 is summed in Table 2.

Additionally, another cohort of 216 unrelated cases suffering from idiopathic AF (115 male cases and 101 female cases, aged 53 ± 9 years) and 600 unrelated healthy people with no family history of AF (319 male persons and 281 female persons, aged 53 ± 7 years) employed as control subjects were enrolled from the Chinese Han-ethnicity population. Clinical investigation revealed that the cases with idiopathic AF were matched in age and sex with the control persons.

### 3.2. A New AF-Causative Locus Mapped on Human Chromosome 7p14.2-p14.3

Via whole-genome screen utilizing microsatellite markers at about 9 cM intervals and a 2-point linkage assay in Family 1 suffering from AF, the greatest LOD score (Zmax) of 3.2602 at a recombination fraction (θ) of 0.00 was preliminarily achieved at marker D7S2252, which would provide significant evidence suggestive of linkage. To refine the chromosomal disease locus, seven additional microsatellite markers at nearby loci surrounding D7S2252 (D7S2515, D7S435, D7S526, D7S656, D7S484, D7S2250, and D7S2209) were used for genotyping the members available from Family 1, with a Zmax of 3.1352 at θ = 0.00 for marker D7S484, and the disease haplotype of Family 1 was deduced with the eight markers (Figure 1). Recombination events occurred in the affected family members III-1 and III-16 at D7S526 and III-8 and IV-7 at D7S2250, which defined a critical disease interval, a novel AF-causing genetic locus, on human chromosome 7p14.2-p14.3 (GRCh38, chr7: 30,909,270–35,338,825), a ~4.89 cM (~4.43-Mb) interval delimited by D7S526 and D7S2250. The two-point LOD scores for the selected eight genetic markers at chromosome 7p14.2-p14.3 utilized to construct the haplotype of Family 1 are shown in Table 3.

### 3.3. Discovery of TBX20 as a New AF-Predisposing Gene

**Figure 5:**
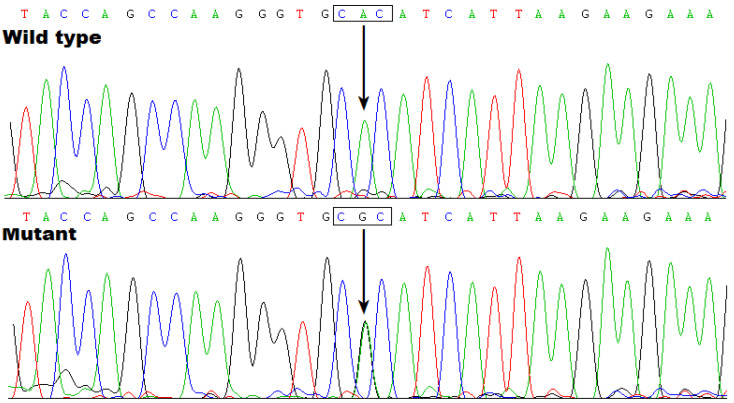
Sequence chromatograms illustrating the *TBX20* c.695A>G mutation in a heterozygous status. An arrow directs to G/A from the proband of Family 1 (mutant) or A/A from a healthy individual of Family 1 (wild type).

As exhibited in Table A1, there exist 55 genes at the located locus between D7S526 and D7S2250, encompassing 18 protein-coding genes and 19 pseudogenes as well as 18 genes encoding non-coding RNAs. Through WES analysis in two members affected by AF (Family 1: III-1 and IV-1) and one healthy member (Family 1: III-2), we found that, at the mapped locus, only the variant chr7:35280609T>C (GRCh37.p13: NC_000007.13), equal to chr7: 35215533T>C (GRCh38.p14: NC_000007.14) or NM_001077653.2: c.695A>G; p.(His232Arg) in *TBX20*, was identified and confirmed via Sanger sequencing assay to co-segregate with AF in Family 1. Specifically, the *TBX20* gene in family 1 has been sequenced in all living members to confirm the presence of the mutation c.695A>G in affected members and the absence of the mutation c.695A>G in unaffected members. The sequence chromatogram of the heterozygous c.695A>G mutation in the *TBX20* gene is exhibited in Figure 5.

**Figure 6:**
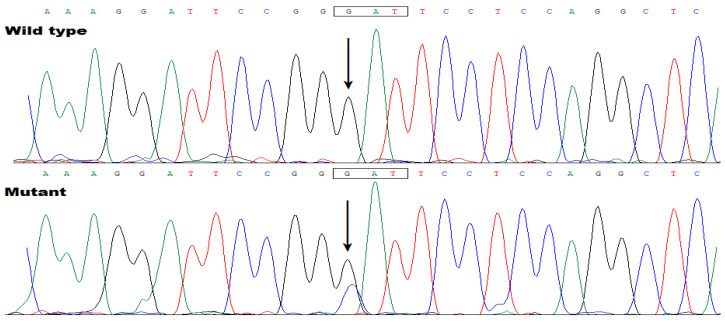
Sequence electropherograms illustrating the *TBX20* c.862G>C mutation in a heterozygous status. An arrow directs to C/G from the proband of Family 2 (mutant) or G/G from an unaffected person (wild type).

In addition, a Sanger sequencing assay of *TBX20* was completed in Family 2 (Figure 3) and in another cohort of 216 index cases with AF as well as 600 unrelated healthy subjects, and a new *TBX20* mutation, NM_001077653.2: c.862G>C; p.(Asp288His), was found to co-segregate with AF in Family 2. The sequence chromatogram of the *TBX20* c.862G>C mutation in a heterozygous status is presented in Figure 6.

The two missense mutations were neither detected in 600 unrelated subjects employed as controls nor published in the population genetics databases of gnomAD and SNP.

### 3.4. Reduced Transactivation of KCNH2 by Mutant TBX20

**Figure 7:**
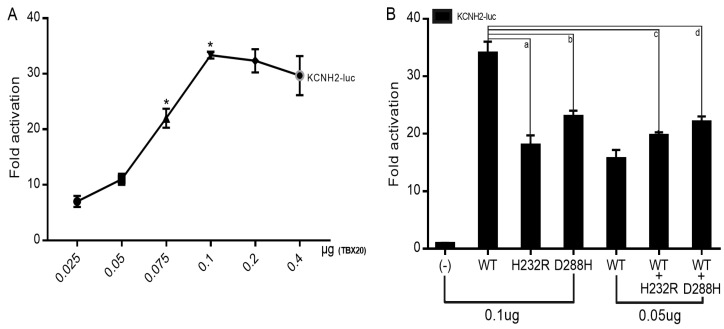
Decreased transactivation effect of TBX20 on *KCNH2* by the mutations. (**A**) In cultured HeLa cells expressing various recombinant plasmids, including different doses (from 25 ng to 400 ng) of wild-type TBX20-pcDNA3.1 (WT), reporter analysis unveiled that TBX20 transcriptionally activated *KCNH2* in a dose-dependent mode from 25 ng to 100 ng. (**B**) In cultivated HeLa cells expressing various recombinant plasmids, including WT, His232Arg-mutant TBX20 (H232R), and Asp288His-mutant TBX20 (D288H), dual-luciferase reporter assay unveiled that both H232R and D288H, singly or together with WT, had significantly decreased transactivation of *KCNH2*. For each expression vector used, reporter assay experiments were repeated three times in triplicate. Here * indicates *p* < 0.05; a, b, c, and d indicate *p* = 0.0005, 0.0010, 0.0003, and 0.0007, respectively, when compared with WT (100 ng).

As indicated in Figure 7A, wild-type TBX20 transactivated the expression of the target gene *KCNH2* in a dose-dependent fashion from 25 ng to 100 ng. As shown in Figure 7B, in the homozygous status, 100 ng of wild-type TBX20 (WT), 100 ng of His232Arg-mutant TBX20 (H232R), and 100 ng of Asp288His-mutant TBX20 (D288H) transactivated *KCNH2* by ~34-fold, ~18-fold, and ~23-fold, respectively (WT vs. H232R: t = 10.4745, *p* = 0.0005; WT vs. D288H: t = 8.5206, *p* = 0.0010); in the heterozygous status, 50 ng of WT plus 50 ng of H232R and 50 ng of WT plus 50 ng of D288H transactivated *KCNH2* by ~20-fold and ~22-fold, respectively (WT vs. WT + H232R: t = 11.9261, *p* = 0.0003; WT vs. WT + D288H: t = 9.2952, *p* = 0.0007).

### 3.5. Diminished Ability of the TBX20 Mutants to Bind the KCNH2 Promoter

**Figure 8:**
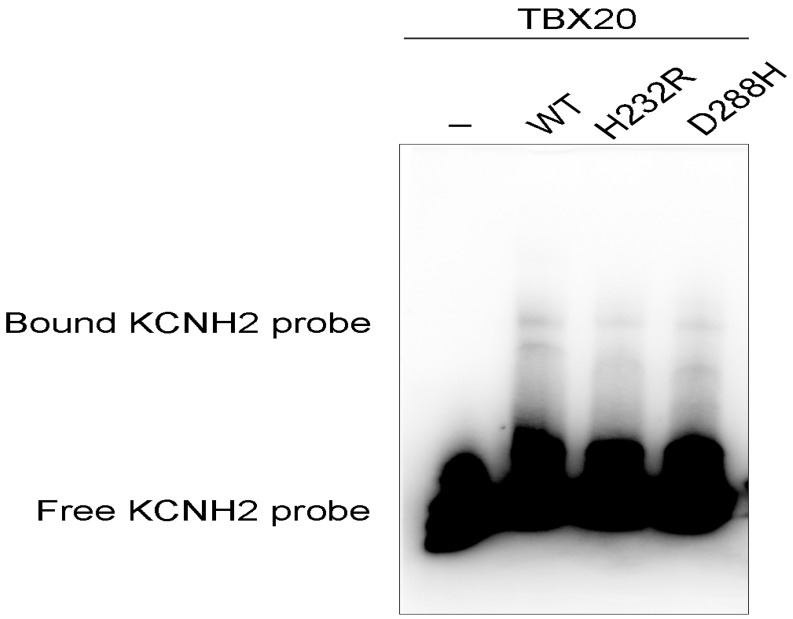
Reduced ability of the mutant TBX20 proteins to bind to the *KCNH2* promoter. Wild-type TBX20 (WT) normally bound the *KCNH2* promoter, while the ability of His232Arg-mutant TBX20 (H232R) or Asp288His-mutant TBX20 (D288H) to bind the *KCNH2* promoter was significantly diminished.

As shown in Figure 8, electrophoretic mobility variation analysis demonstrated that wild-type TBX20 (WT) bound properly the biotinylated *KCNH2* promoter (DNA probe) to form complexes, whereas the ability of His232Arg-mutant TBX20 (H232R) or Asp288His-mutant TBX20 (D288H) to bind the *KCNH2* promoter was significantly diminished when compared to that of the WT. Particularly, the ability of H232R to bind the *KCNH2* promoter was reduced to an undetectable level.

### 3.6. Subcellular Distribution of TBX20 Mutants

**Figure 9:**
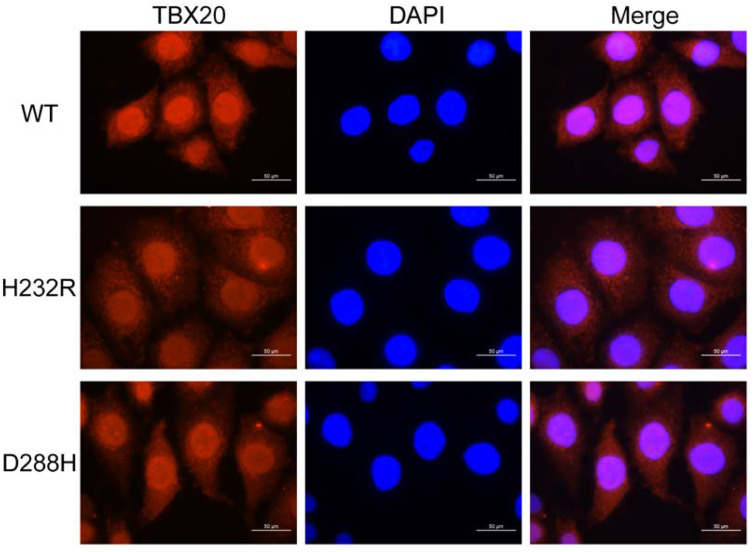
Normal intracellular distribution of the TBX20 mutants. Subcellular distribution of wild-type TBX20 (WT), His232Arg-mutant TBX20 (H232R), and Asp288His-mutant TBX20 (D288H) was observed in cultured HeLa cells by immunofluorescence. The HeLa cells expressing WT, H232R, or D288H were stained by utilizing anti-FLAG (red) and DAPI (blue). Magnified cellular images with FLAG and DAPI stained and merged cellular images were shown. Cellular immunofluorescent images demonstrated that WT, H232R, or D288H was distributed predominantly in the cellular nuclei. Bar = 50 μm.

Wild-type TBX20 (WT) was normally distributed to the cellular nucleus (Figure 9). Like the WT, both His232Arg-mutant TBX20 (H232R) and Asp288His-mutant TBX20 (D288H) showed normal intracellular distribution, with each mutant localized predominantly to the cellular nucleus (Figure 9).

## 4. Discussion

Herein, through whole-genome genotyping with polymorphic DNA markers and linkage and haplotype analyses in a family suffering from AF, a new AF-causing locus was located at human chromosome 7p14.2-p14.3. An exome-wide sequencing assay revealed that, at the defined locus, the mutation in the *TBX20* gene, NM_001077653.2: c.695A>G; p.(His232Arg), was solely co-segregated with AF in the entire family (Family 1). Additionally, a Sanger sequencing assay of *TBX20* in another family suffering from AF uncovered a novel mutation, NM_001077653.2: c.862G>C; p.(Asp288His), which was co-segregated with AF in the entire family (Family 2). The two *TBX20* mutations were neither found in the 1200 referential chromosomes nor published in the databases of gnomAD and SNP. Functional studies uncovered that the two mutant TBX20 proteins both showed significantly reduced transactivation on the target gene *KCNH2* (a well-established AF-causing gene) and decreased ability to bind the promoter of *KCNH2*, with no effect on the nuclear distribution of TBX20. These findings define a novel AF-predisposing locus at chromosome 7p14.2-p14.3 and convincingly indicate genetically compromised *TBX20* as a new gene contributing to AF.

Members of the large T-box (TBX) gene family, encompassing TBX20, TBX5, TBX1, TBX18, TBX3, and TBX2, are identified as crucial players that act in normal cardiac organogenesis, including cardiac lineage determination at early stage, valvuloseptal morphogenesis, the chamber specification of the heart, and the diversification of the cardiac conduction system during embryogenesis in vertebrates [88]. These TBX genes code for a family of TBX-containing transcription factors, which recognize and bind to the so-called T-half sites (5′-AGGTGTGA-3′) existing in the promoters/enhancers of downstream genes, and mediate transactivation or the transcriptional repression of target genes, hence exerting complex temporal–spatial regulation in the developing heart [88,89]. Additionally, the TBX domain is also responsible for the interaction with other transcriptional factor partners, histone-modifying enzymes, and chromatin remodeling complexes involved in the hierarchies of transcriptional mediation [90]. In human beings, *TBX20* is located at chromosome 7p14.2, coding for a protein with 447 amino acids [89]. Previous experiments have revealed that TBX20 is amply expressed in the developing and adult hearts [91] and transcriptionally activates an array of target genes expressed amply in the heart, including *NPPA* (*ANP*), *GJA5* (*Cx40*), *GJC1* (*Cx45*), and *KCNH2*, singly or in synergy with its cooperative partners, including TBX5, GATA4, GATA5, and NKX2-5 [76,92,93]. Deleterious mutations in the genes of *NPPA* [94], *GJA5* [95,96,97], *GJC1* [65], *TBX5* [98,99,100], *GATA4* [101,102,103], *GATA5* [104,105], *NKX2-5* [106,107,108], and *KCNH2* [76] have been discovered to be accountable for AF. In this research, two new pathogenic mutations in *TBX20* were uncovered to be responsible for AF. These results strongly indicate that *TBX20* dysfunction predisposes to AF, probably by lowering the expression of downstream genes.

The increased susceptibility to AF in patients harboring a functionally defective *TBX20* allele may be partly attributed to structural and electrophysiological abnormalities of the heart [76,90,91,93,109]. Previous investigations have demonstrated that *TBX20* plays important roles in cardiac embryonic development, internal homeostasis, the function of adult hearts, and pathophysiological adaptation, including its important roles in cardiac electrophysiology [91]. The heart conduction system is a group of complex special structures and cells in the heart, including the sinus and atrioventricular nodes as well as the atrioventricular bundle, left and right bundle branches, and Purkinje fiber mesh, which in a spatial and temporal way accurately regulates the electric pulse conduction, inducing coordinated heart rhythm and synchronous heart contraction to maintain normal blood circulation [91]. Moreover, cardiac working myocytes also play key roles in the myocardial propagation of electrical pulses [91]. Congenital defects or the dysregulated homeostasis of the conduction system can lead to cardiac conduction dysfunction, triggering life-threatening arrhythmias in children and adults and can significantly increase the risk of death in patients [91,101,110,111,112,113,114]. Genome-wide association analyses in cases affected by arrhythmias showed that abnormal electrocardiograms were closely related to cardiac structural proteins, connexins, ion channels, and some key transcription factors that function in the specialization, differentiation, and homeostasis of the heart conduction system, encompassing TBX20 [114]. Although TBX20 was not initially recognized to be involved in the development of the conduction system, whole-genome association analyses associated variations within *TBX20* with a long QRS duration, implicating these regions of TBX20 in transcriptional regulation. These results reveal that TBX20 is involved in the development/maintenance of the conduction system and in the regulation of myocardial conduction [115,116]. TBX20 most likely coordinates and maintains the spatiotemporal regulation of the development and function of the heart, including its conduction system, through multiple-gene regulatory networks [91]. Studies by Shen and colleagues [117] and Sakabe and coworkers [118] showed that mice with conditional knockout of the *Tbx20* gene in adult cardiomyocytes presented with cardiac expansion, a loss of contractile function, decreased heart conduction velocity, and severe arrhythmias. Chromatin immunoprecipitation and enhancer analysis revealed that TBX20 had a wide range of direct target genes regulating cardiac rhythm function [118], and mutations in these downstream target genes mainly caused human inherited ion channelopathies [117,119,120,121]. Importantly, a recent investigation further showed that TBX20 could selectively regulate the expression of KCNH2 [75]. *KCNH2* codes for Kv 11.1 (hERG), the pore-forming α subunit of a rapidly activated delayed-rectified K^+^ channel (with the auxiliary β subunit encoded by *KCNE2*), and the currents produced by these rapidly activated delayed-rectified potassium channels are among the main currents responsible for myocardial repolarization [75,122,123,124]. Further studies revealed that the human TBX20 Arg311Cys mutation (found in families with long QT syndrome) can cause the loss of the transactivation function of TBX20, resulting in decreased expression levels of hERG and a decreased inward rectification current, resulting in prolonged action potential [75]. It is generally understood that triggered (ectopic) activity and re-entry are two major arrhythmogenic mechanisms underlying AF, and triggered activity may be induced by early afterdepolarization that is caused by prolonged action potential [125,126]. Previous studies have found that mutations in *KCNH2* or *KCNE2* can also cause AF [76,77,78,127,128], in addition to long QT syndrome, ventricular arrhythmia, and sudden death [129,130,131,132]. Moreover, TBX20 can also modulate the expression of CAMK2D, CACNA1A, RYR2, ATP2A2, KCND3, CACNA1C, PLN, and KCND2, which underscores the pivotal role of TBX20 in maintaining the normal electrophysiology of the heart [91,117,125,126]. These findings indicate that *TBX20* gene mutations increase susceptibility to AF by modifying the structural and electrophysiological properties of the heart.

Notably, in humans, multiple *TBX20* mutations have been discovered to give rise to various congenital heart defects, encompassing atrial/ventricular septal defects, Fallot’s tetralogy/pentalogy, common atrioventricular canals, double outlets of the right ventricle, aortic coarctation, patent ductus arteriosus, abnormal pulmonary vein connections, and cardiac valve malformation, as well as dilated cardiomyopathy [133,134,135,136]. In the present study, three patients harboring a *TBX20* mutation (members II-8 and III-13 in Family 1 and member II-7 in Family 2) also had congenital atrial septal defects in addition to AF. These results underscore the critical roles of *TBX20* in human cardiac development and structural remodeling, showing that that *TBX20* mutations predispose to congenital heart disease and dilated cardiomyopathy.

## 5. Conclusions

Conclusively, in the current study, a novel AF-predisposing locus is mapped to human chromosome 7p14.2-p14.3, and, within this locus, *TBX20* is identified as a new AF-predisposing gene. These findings not only provide important insight into the genetic mechanism underpinning AF but also imply clinical significance for the individualized management of patients affected by AF.

## Figures and Tables

**Table 1:** Phenotypic characteristic profiles of the living members with atrial fibrillation from Family 1.

Subject Information			Phenotype	Electrocardiogram			Echocardiogram	
Identity (Family 1)	Sex	Age (Years)	AF (Classification)	Heart Rate (bpm)	QRS Interval (ms)	QTc (ms)	LAD (mm)	LVEF (%)
II-1	M	70	Permanent	72	95	407	46	58
II-3	M	68	Permanent	131	116	466	40	61
II-8	F	63	Permanent	153	137	467	43	56
II-10	F	61	Permanent	82	90	432	39	64
III-1	M	47	Permanent	80	101	405	38	66
III-8	F	43	Persistent	84	101	418	39	62
III-9	M	41	Persistent	95	97	377	37	65
III-13	M	39	Persistent	87	102	451	36	67
IV-1	F	22	Paroxysmal	100	88	447	33	63
IV-4	M	20	Paroxysmal	96	84	430	30	64

AF: atrial fibrillation; M: male; F: female; LAD: left atrial diameter; LVEF: left ventricular ejection fraction; LVEF: left ventricular ejection fraction; QTc: corrected QT interval.

**Table 2:** The phenotypic characteristic information of the AF members alive from Family 2.

Subject Information			Phenotype	Electrocardiogram			Echocardiogram	
Identity (Family 2)	Sex	Age (Years)	AF (Classification)	Heart Rate (bpm)	QRS Interval (ms)	QTc (ms)	LAD (mm)	LVEF (%)
II-2	F	65	Permanent	94	101	469	43	55
II-3	M	63	Permanent	66	88	427	39	63
II-7	M	58	Permanent	92	132	461	40	65
III-4	F	41	Persistent	131	94	434	38	60
III-7	M	40	Persistent	73	78	415	37	64
III-10	F	38	Paroxysmal	93	105	433	36	62
IV-4	M	18	Paroxysmal	89	94	481	32	66

AF: atrial fibrillation; M: male; F: female; LAD: left atrial diameter; LVEF: left ventricular ejection fraction; QTc: corrected QT interval.

**Table 3:** Two-point scores of logarithms of odds (LOD) for the selected eight microsatellite markers at human chromosome 7p14.2-p14.3.

Marker	LOD Scores at Recombination Fraction θ =						
	0.00	0.01	0.05	0.10	0.20	0.30	0.40
D7S2515	(‒∞)	1.3796	1.8468	1.8462	1.4996	0.9661	0.3777
D7S435	(‒∞)	0.1973	0.7540	0.8709	0.7801	0.5341	0.2366
D7S526	(‒∞)	−1.4378	−0.6495	−0.2873	0.0189	0.1000	0.0743
D7S2252	3.2602	3.1991	2.9495	2.6244	1.9262	1.1641	0.4210
D7S656	2.4082	2.3646	2.1855	1.9507	1.4413	0.8808	0.3321
D7S484	3.1352	3.0785	2.8468	2.5452	1.8971	1.1817	0.4386
D7S2250	(‒∞)	0.7841	1.2783	1.3139	1.0507	0.6237	0.1789
D7S2209	(‒∞)	1.6784	2.1357	2.1202	1.7332	1.1382	0.4454

LOD: logarithm of odds.

## Data Availability

All data are provided in this manuscript as well as Appendix A Table A1.

## Appendix A

**Table A1:** A complete list of all 55 genes at the chromosomal region delimited by markers D7S526 and D7S2250.

**AQP1**	**GHRHR**	LOC105375222(lncRNA)	LOC105375221 (lncRNA)	**ADCYAP1R1**
LOC124901821 (pseudo)	LOC107986781 (lncRNA)	LOC107986782 (lncRNA)	**NEUROD6**	LOC105375224 (lncRNA)
**ITPRID1**	**PDE1C**	**PPP1R17**	SNX2P2 (pseudo)	LOC100130673 (pseudo)
SLC25A5P5 (pseudo)	**LSM5**	**AVL9**	DPY19L1P1 (pseudo)	ZNRF2P1 (pseudo)
MIR550A2 (miRNA)	MIR550B2 (miRNA)	LINC00997 (lncRNA)	DPY19L1P2 (pseudo)	**KBTBD2**
RP9P (pseudo)	**FKBP9**	RNU6-388P (pseudo)	**NT5C3A**	RPS29P14 (pseudo)
RN7SL505P (pseudo)	LOC124901610 (lncRNA)	**RP9**	**BBS9**	RNA5SP229 (pseudo)
LOC100421336 (pseudo)	LOC124901611 (lncRNA)	FLJ20712 (lncRNA)	LOC124901612 (lncRNA)	LOC124901613 (lncRNA)
**BMPER**	RNU6-438P (pseudo)	NPSR1-AS1 (lncRNA)	**NPSR1**	RPL7P31 (pseudo)
NCAPD2P1 (pseudo)	RN7SL132P (pseudo)	**DPY19L1**	MIR548N (miRNA)	LOC102724723 (lncRNA)
LOC105375228 (lncRNA)	DPY19L2P1 (pseudo)	S100A11P2 (pseudo)	**TBX20**	LOC401324 (lncRNA)
